# Silicon seed inoculation enhances antioxidants, physiology and yield of hybrid maize under heat stress

**DOI:** 10.1186/s12870-025-06399-9

**Published:** 2025-04-02

**Authors:** Sajid Munawar, Rao Muhammad Ikram, Reimund P. Roetter, Ijaz Hussain, Muhammad Afzal, Abdel-Halim Ghazy, Saeed Ahmad, Muhammad Habib-ur-Rahman

**Affiliations:** 1 Department of Agronomy, Faculty of Agriculture and Environmental Sciences, MNS University of Agriculture, Multan, Pakistan; 2 Tropical Plant Production and Agricultural Systems Modelling (TROPAGS), University of Goettingen, Grisebachstr. 6, 37077 Goettingen, Germany; 3 Campus Centre of Biodiversity and Sustainable Land Use (CBL), University of Goettingen, Buesgenweg 1, 37077 Goettingen, Germany; 4 Plant Production Department, College of Food and Agriculture Sciences, King Saud University, Riyadh, 2460, 11451 Saudi Arabia; 5 Department of Agricultural and Biological Engineering, University of Florida, Gainesville, FL USA

**Keywords:** Physiological and antioxidant mechanism, Crop growth, Heat stress mitigation, Sustainable production, Arid to semiarid climate

## Abstract

**Background:**

Heat stress, next to drought, is one of the major constraints to maize crop growth, development and sustainable yield in the tropics and sub-tropics, particularly in arid and semi-arid climatic regions. Hence, there is a dire need to explore strategies that alleviate adverse effects of heat stress. In this regard, silicon (Si) is an important plant nutrient which may support crop in alleviating heat stress-induced damages by modulating plant defense mechanisms. The aim of the study was to explore the potential role of Si for inducing heat tolerance in hybrid maize. Yet, to date, limited knowledge is available on how Si modulates plant defense mechanisms to induce heat tolerance in maize crop.

**Methods:**

Two maize hybrids were adopted for field experiment (heat tolerant and sensitive selected from a pot experiment study) on the basis of traits performance through screening in the glasshouse. Six maize hybrids were tested at different heat stress levels (T_1_ = control; T_2_ = 40 °C ± 3 and T_3_ = 45 °C ± 3 for a period of 6 h per day) at six leaf growth stage (V6) in the glasshouse. Secondly, a field experiment was conducted to evaluate the effect of Si seed inoculation [Si_0_ = 0.0 mM (control); Si_1_ = 3.0 mM (recommended); Si_2_ = 6.0 mM] on physiology, growth, antioxidants activity and yield traits of two selected maize hybrids; H_1_ = AA-9633 (heat sensitive); H_2_ = YH-5427 (heat tolerant) under heat stress conditions (HS_0_ = control (without heat stress); HS_1_ = heat stress at pollination stage- 65 days after sowing for a period of 8 consecutive days).

**Results:**

The field study results showed that maize hybrid “YH-5427”, a prior rated as heat tolerant, produced higher cob length, number of grains per cob, thousand grain weight and grain yield through improved photosynthetic rate, stomatal conductance, water use efficiency, activity of superoxide dismutase, peroxidase and catalase with the seed inoculation of Si (6.0 mM) under heat stress conditions. However, heat sensitive hybrid (AA-9633) produced reduced grain yield (9.26%) and yield components as attained by YH-5427 with the seed inoculation of Si (6.0 mM) under heat stress conditions.

**Conclusion:**

Maize hybrid YH-5427 with Si seed inoculation (6 mM) is a promising option to maintain relatively high maize grain yield (t ha^− 1^) under heat stress conditions.

## Background

Climate change has become a major threat for sustainable agricultural production globally due to marked shifts in temperature regimes, rainfall patterns and frequency of extreme events [1–4]. Climate extreme conditions particularly heat and related plant stresses are expected to decrease maize production threating food security, especially in semi-arid /water scarce regions [5, 6]. The crop productivity in a particular region is determined by its existing climatic conditions i.e. temperature, precipitation, and solar radiations [7–9]. Therefore, a minor change in the weather conditions poses severe negative impacts on crop productivity [10, 11]. Pakistan is ranked as 5^th^ most badly affected countries by climate change [12, 13]. The frequency and intensity of compound climate extreme events have increased significantly. A recent example is the record-breaking heatwaves observed (March to May, 2022) in Southern Pakistan since 1901 Temperatures recorded constantly ranged 3 °C to 8 °C above the long-term average that breach the historic records in both India and Pakistan [14]. Extreme climate events, and rising temperature pose significant constraints for crop productivity globally, including Pakistan [15–17]. Among these, climate induced-heat stress is a major threat, adversely affecting the growth, phenology, physiology and yield contributing traits of maize, particularly in arid and semi-arid regions [18–20].

To mitigate the adverse effects of heat stress on maize production, various strategies, including genetic and agronomic approaches, have been explored [21, 22]. While these strategies have shown varying degrees of success, they often require extensive breeding efforts, high input costs, or long-term implementation. In contrast, silicon (Si) has gained increasing attention for its role in enhancing plant resilience under abiotic stress conditions by improving physiological and biochemical responses and mechanisms. Although substantial evidence supports the positive effects of Si in mitigating drought, salinity, and heavy metal stress, its application in heat stress management remains largely underexplored. In particular, silicon seed inoculation has not been widely investigated as a viable approach for inducing heat tolerance in maize hybrids. Given the rising frequency and intensity of heat stress events due to climate change, there is an urgent need to evaluate Si seed inoculation as a cost-effective, sustainable, and easily adoptable strategy for enhancing heat tolerance in maize hybrids.

Heat stress negatively impacts the physiological processes in plants by disrupting enzymatic activity and other cellular functions [22–24]. For instance, the photosynthetic rate slow down at higher temperatures, reducing energy production and ultimately leading to a significant reduction in crop growth and yield [25–27]. Extreme heat can also reduce pollen fertility and disrupt the timing of flowering, resulting in poor pollination and lower crop yields [8]. Furthermore, heat stress affects the rate, intensity and duration of damage in chloroplasts and mitochondria, harming the electron transport chain, plant cells, proteins, lipids, primary and secondary metabolites [23]. The activities of enzymatic and non-enzymatic antioxidant compounds including catalase, peroxidase, phenol, glutathione and ascorbic acid are decreased due to increased production of reactive oxygen species (ROS) under heat stress conditions, resulting in lower crop yields [28, 29]. It has also been reported that heat stress can reduce crop yield by up to 45% [30].

Maize (*Zea mays* L.) is the third most important cereal crop in Pakistan, following wheat and rice [31]. It is widely cultivated in both spring and autumn seasons for animal feed and as a raw material for industries. However, spring-sown maize productivity faces a severe challenge due to high temperature in April and May, particularly during reproductive phases and grain development [32]. The reproductive stage is highly sensitive to heat stress, leading to a significant reduction in maize productivity [32–34]. In Pakistan, maize sown in the spring season reaches its reproductive phase in April and May, a period of typically high temperatures and more frequent heatwaves, as observed in March, 2022. Consequently, spring-sown maize experiences substantial negative effects from heat stress during these critical months, impacting yield and overall crop health. It has been anticipated that maize yield may decrease by 8–14% due to increase in globe temperature by 2 °C [35, 36]. Several studies have also indicated that high temperature negatively affect the growth and yield traits of maize [37–39]. A huge decline in photosynthetic activity and grain yield has been reported when heat stress occurs at the anthesis and silking stages [40, 41]. Higher temperatures also lead to a significant reduction in the size, weight and quality of maize grains [42]. Therefore, it is essential to minimize heat stress-induced damages in maize using cumulative strategies, including heat tolerant varieties, and seed inoculation with beneficial elements. In this context, silicon (Si) seed inoculation might be a sustainable strategy to alleviate the adverse effects of heat stress in maize [43].

Silicon seed inoculation is beneficial and cost effective for inducing heat tolerance in plants [44, 45]. It plays a crucial role in decreasing oxidative stress in plants by modulating physiological and antioxidant activities under heat stress conditions [46–48]. In this way, Si helps heat-stressed plants maintain physiological processes even under severe heat stress [49, 50]. Several studies have shown that Si seed inoculation improves the seed germination, photosynthetic performance, nutrient absorption, secondary metabolites production and ultimately leads to greater total dry matter accumulation and crop yield [51, 52]. Additionally, Si seed inoculation has significantly improved the growth and yield of various crops such as salvia, date palm, tomato, cucumber, rice, barley and strawberry under heat stress [18, 53].

Although literature is available on other inoculation agents, studies specifically focusing on the role of Si as a seed priming agent remain underexplored in the context of inducing heat tolerance in maize hybrids. Most existing research emphasizes genetic approaches or other agronomic practices without adequately addressing the potential benefits of Si seed inoculation under heat stress conditions. However, increasing evidence from other studies highlights the role of Si in mitigating abiotic stresses, suggesting that silicon seed inoculation may have a crucial role in alleviating the damaging effects of heat stress in maize. Limited studies have been conducted to assess the underlying mechanisms of Si seed inoculation in improving physiological processes, antioxidants activity, as well as in alleviating heat stress-induced damages in maize. Therefore, the present study hypothesized that Si seed inoculation may enhance physiological processes, antioxidants activity and consequently yield components of maize hybrids under heat stress conditions. The main goal of the present study is to evaluate the potential impacts of different levels of Si seed inoculation on the morpho-physiological traits and antioxidant activity of maize hybrids under heat stress.

## Materials and methods

### Experimental site, soil and Climatic conditions

**Fig. 1:**
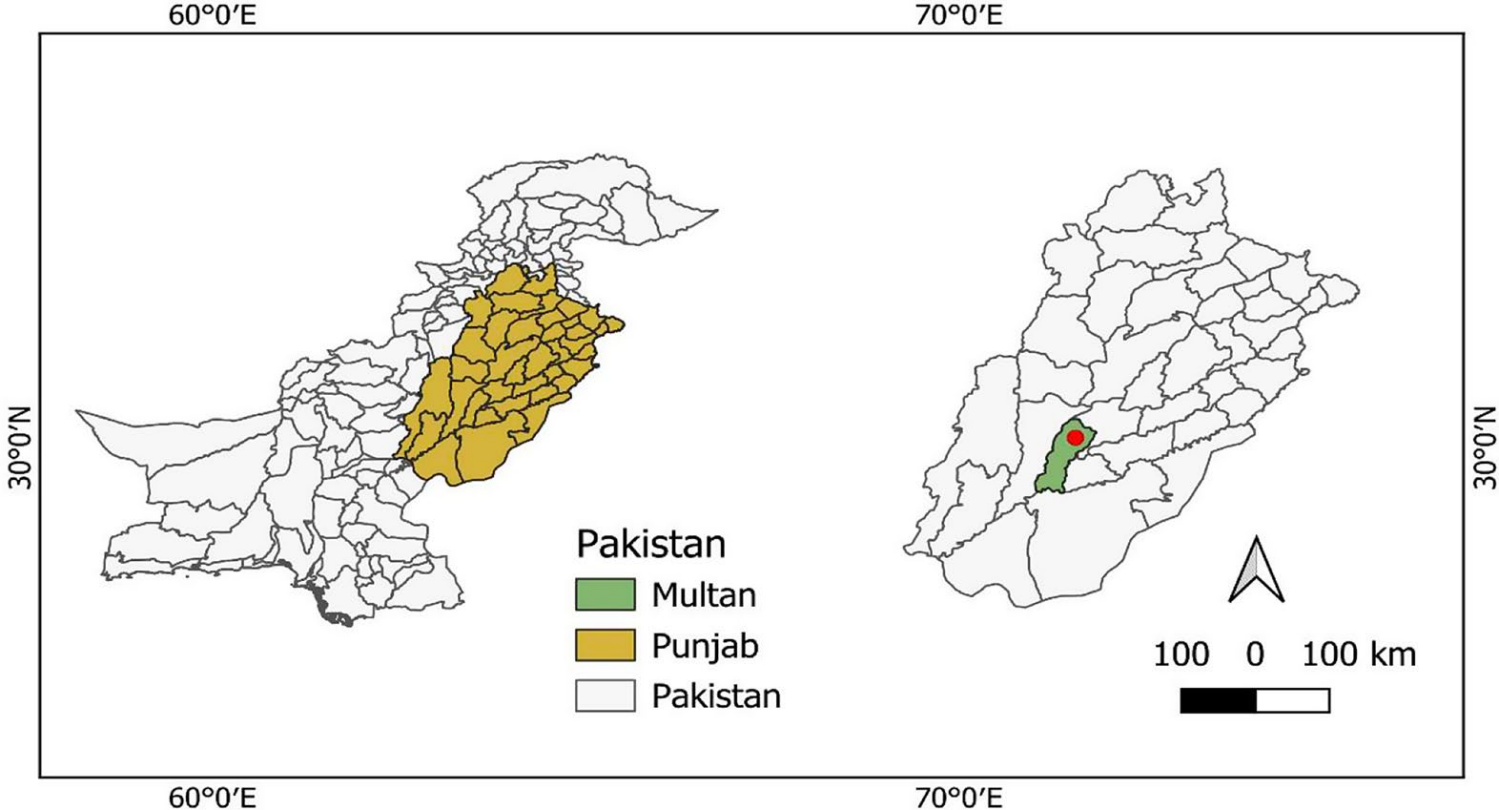
The experimental study site is located in an arid climatic region. The red spot precisely indicates the experimental site in Multan

**Fig. 2:**
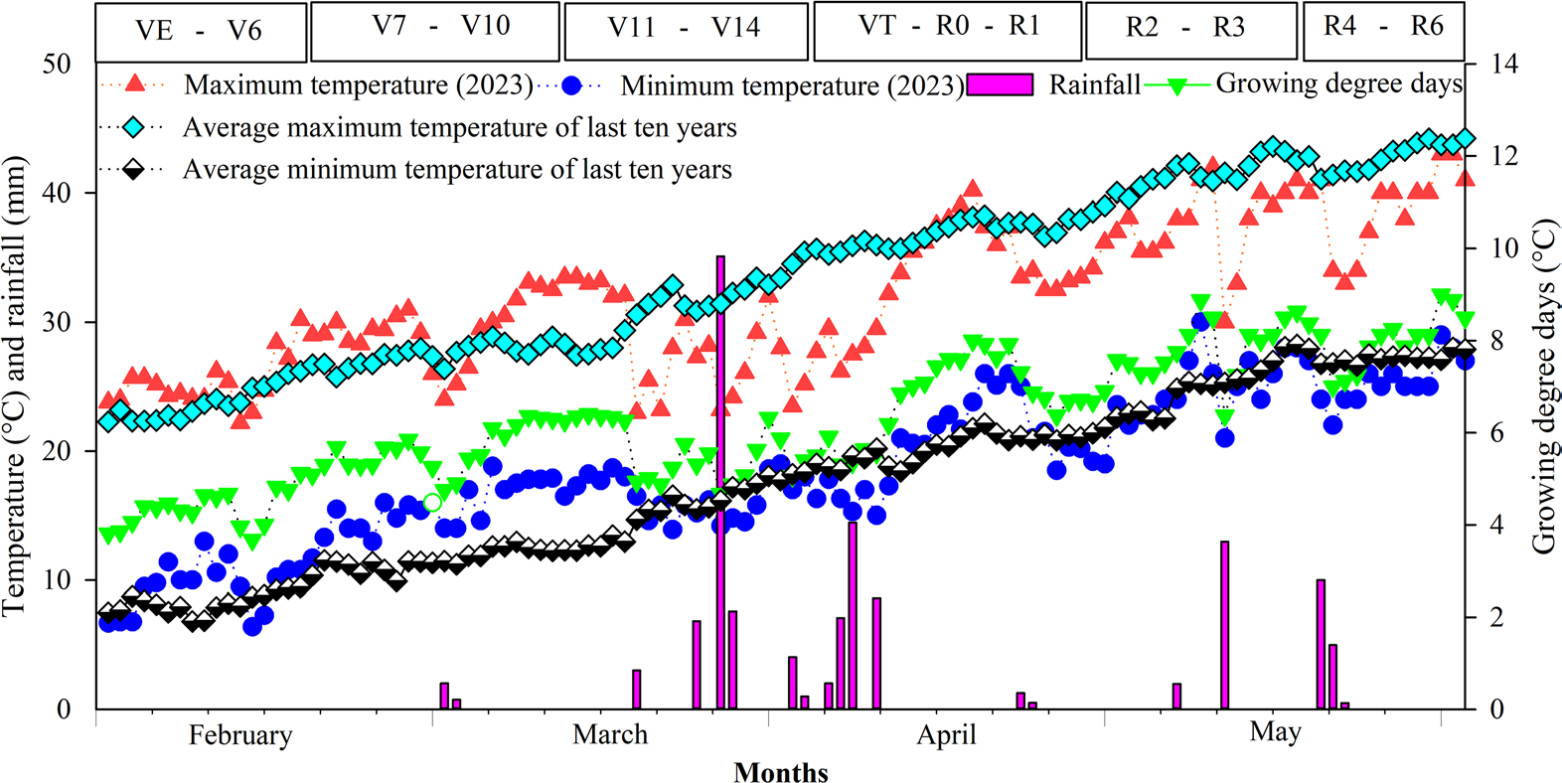
Daily climate data (rainfall, maximum and minimum temperatures, and growing degree days [GDDs]) at the experimental site during the 2023 growing season, along with maize growth stages (Ritchie & Hanway, 1993). The average maximum and minimum temperatures for the decade (2012–2021) are also presented, while data for 2022 was excluded due to extreme climate events (heatwaves)

The field experiment was conducted at the research area of MNS-University of Agriculture, Multan, Pakistan (Fig. 1, map of the study location) during the spring season of 2023. While the pot experiment was conducted under controlled glasshouse conditions. The experimental site is characterized by an under arid climate (sub-tropical) with high temperatures during the summer months and moderate rainfall during the monsoon season (July-August). Over the past decade, there has been significant variation in maximum temperatures, particularly with the heatwaves in 2022 that resulted in the hottest March and April in the subcontinent since 1901. The experimental soil is sandy loam in texture, consisting of 45% sand, 30% silt and 25% clay with pH value of 7.8, electrical conductivity of 1.43 dS m^− 1^, organic matter content (0.62%), extractable potassium (188.3 mg kg^− 1^), available phosphorus (5.11 mg kg^− 1^) and total available nitrogen of 73.4 mg kg^− 1^. The daily weather data for the entire crop period (February-June) was collected from an Automatic Weather Station installed at the experimental research site (Fig. 2). Historical weather data for the study region was collected from Pakistan Meteorological Department (PMD), Islamabad. The total rainfall during the growing season of the maize crop was 148 mm. The highest temperature (46 °C) was recorded in May, 2023 (peak growing season and reproductive phases). In contrast, the minimum temperature of 6.4 °C was recorded in February (early vegetative phase of maize development). Daily maximum air temperature (Tmax) and minimum temperature (Tmin) were also used to calculate growing degree days (GDD) above a threshold temperature (TT) in terms of degrees days (DD). Degrees days were calculated with an Eq. (1) that determines DD as the difference between the daily mean temperature and the threshold temperature (TT) specific for maize crop.

1 $$\text{DD}\:\left(^{\circ}\text{C}\:\text{days}\right)=\sum\limits_{{\rm{i}} = {\rm{dh}}}^{{\rm{i}} = {\rm{ds}}} {\left[ {\left\{ {{{{\rm{Tmax}} + {\rm{Tmin}}} \over 2}} \right\} - {\rm{TT}}} \right]} $$

Where, DD (ºC days) accumulation is the accumulative degrees days for specific phase, “ds” is the date of sowing, “dh” date of maturity, TT is threshold temperature which was considered as 10 ºC for maize crop to compute degree days (54). In this case, if [(Tmax + Tmin)/2] < TT, or [(Tmax + Tmin)/2] = TT then DD was considered equal to zero.

### Experimental design and treatments

Two experiments (a glasshouse experiment and a field experiment) were conducted under arid climatic conditions. The pot experiment followed a completely randomized design (CRD) with four replications and was conducted in a glasshouse. It included three heat stress levels (T_1_ = control; T_2_ = 40 °C ± 3 and T_3_ = 45 °C ± 3) applied for six hours per day (10:00 am – 4:00 pm) and six maize hybrids (FH-1046, AA-9633, FH-949, DK-7024, YH-5427 and NK-6654). Seeds of six selectedmaize hybrids were initially grown in sandy loam soil under optimal conditions until plants reached the six-leaf stage (V6). Heat stress was then imposed for six hours per day over an eight-day period (25–30 days after emergence) under controlled glasshouse conditions. Temperature levels were maintained using a controlled heating system and continuously monitored with temperature sensors installed in the glasshouse. The thermometers were positioned horizontally on wooden stands, elevated just above the crop canopy to ensure accurate temperature monitoring. A control treatment was maintained under optimal conditions for comparison with heat-stressed seedlings. Following the heat stress period, plants were allowed to recover under normal conditions, and data on maize seedling growth traits were recorded 35–40 days after emergence using standard protocols.

**Fig. 3:**
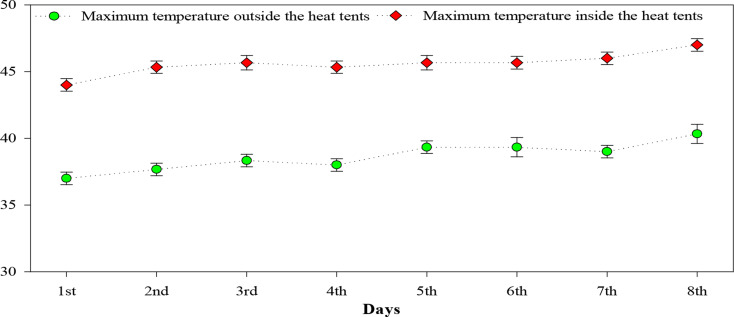
Comparison of ambient maximum temperature (outside the heat tents) and induced high temperature (inside the heat tents) during the 8-day heat stress period at the field experimental site under arid climatic conditions

Following the pot experiment, a field experiment was conducted using a randomized complete block design (RCBD) with split-split plot arrangement and four replications. The plant to plant and row to row spacing were maintained at 30 cm and 75 cm, respectively. The main experimental plots were assigned two heat levels: HS_0_ (control, no heat stress) and HS_1_ (heat stress at reproductive stage: VT-R0-R1, Ritchie scale, 45 ± 3 °C. Sub plots were designated for two maize hybrids: H_1_ (AA-9633, heat sensitive) and H_2_ (YH-5427, heat tolerant). The sub-sub plots included three levels of silicon application (Si_0_ = 0 mM; Si_1_ = 3 mM; Si_1_ = 6 mM). Heat stress was imposed at pollination stage (65 DAS) using heat tents [54], which were covered with the transparent polythene sheet for 6 h per day (10:00 am – 4:00 pm), over 8 days (Fig. 3).

Heat tents were not installed on the control plots. To prevent air stagnation and excessive temperatures beyond the target range (45 ± 3 °C), one side of polythene sheet was periodically opened. The polythene sheets were removed at night to allow natural cooling and prevent excessive moisture buildup within the heat tents. Temperature was recorded inside and outside the polythene sheet through temperature sensors to compare the heat stress levels on the maize hybrids. Moreover, four temperature sensors were installed at plant canopy heights in center of the heat tents and near the corners to detect the temperature variations. Following the heat stress period, the polythene sheets were removed and plants were allowed to recover under normal conditions. The data on morpho-physiological traits and antioxidant activities and yield components was recorded using standard protocols and procedures.

### Crop husbandry

The maize crop was sown during spring season. A fine seedbed was prepared through three ploughings, followed by two planking’s using cultivator. Ridges were formed with a tractor mounted ridge planter and seeds were manually sown after inoculating both maize hybrids with different levels of silicon. The crop was sown on ridges by using seed rate of 30 kg ha^− 1^, with a plant population of 44,444 plants ha^− 1^. Heat stress was applied at reproductive stage (pollination stage: VT-R0-R1, 65 days after emergence) using heat tents. However, the sheets were partially removed to prevent excessive heat accumulation and ensure adequate aeration. All agronomic practices were maintained uniformly throughout the growing season. The maize crop was harvested after the ripening of cob kernels. Further, details on crop management practices and cultural operations required for optimal spring maize cultivation are provided in Table 1.

**Table 1:** Crop management practices and cultural operations during crop growing season for the spring sown maize crop in the field

Crop management practices	Date	Rate of application	Machinery used for different operations
Crop: Maize Cultivar: AA-9633; YH-5427			
Field Preparations: 2–3 ploughings and planking	08-02-2023		Cultivator Laser land Leveler Ridger/furrower
Sowing time	10-02-2023		
Sowing method: Ridge sowing			Dibbler
Seed rate:		30 kg ha^− 1^	
Row-Row distance: 75 cm Plant-Plant distance: 30 cm Sowing depth of seed: 3–5 cm			
Herbicides application: Pre-mergence herbicides Pendimethalin)		330 g/L^− 1^*A.I.* ha^− 1^	Sprayer
Post emergence herbicides [Atrazine (50 g/L) + Mesotrine (500 g/L) Formulation: SC (Suspension concentrate)			Sprayer
Fertilizer Application: First application: Phosphorus (P); Potassium (K) 1^st^ half dose of Nitrogen (N)	08-02-2023	*N* = 227 kg ha^− 1^ *P* = 120 kg ha^− 1^ K = 100 kg ha^− 1^ 113 kg ha^− 1^	Broadcast method and incorporated
2^nd^ half dose of remaining N	16-02-2023	57 kg ha-1	Fertigation
3^rd^ dose of remaining N	21-03-2023	57 kg ha-1	Fertigation
Irrigation application:			
1^st^ Irrigation (sowing)	10-02-2023		
2^nd^ Irrigation (VE)	16-02-2023		
3^rd^ Irrigation (VE - V6)	27-02-2023		
4^th^ Irrigation (V6)	21-03-2023		
5^th^ Irrigation (V6 - V10)	04-04-2023		
6^th^ Irrigation (V11 - V14)	08-04-2023		
7^th^ Irrigation (VT - R0 - R1)	15-04-2023		
8^th^ Irrigation (VT - R0 - R1)	28-04-2023		
9^th^ Irrigation (R2 - R3)	12-05-2023		
10^th^ Irrigation (R4 - R6)	22-05-2023		
Total water applied:		750 mm	
Insecticides application: Coragen: (Chlorantraniliprole)		20 SC (125 ml *A.I.* ha^− 1^)	Sprayer
1^st^ application	17-2-2023		
2^nd^ application	27-02-2023		
3^rd^ Granular insecticide: Fertera (Chlorantraniliprole)	19-03-2023	4 kg ha^− 1^	
Harvesting			Combine harvester

*A.I. = active ingredient; Irrigations were applied on crop growth stages described by Ritchie and Hanway, 1993

### Observations and data collection in glasshouse

Data on various growth traits of maize seedlings grown under glasshouse was recorded at 40 days after sowing (DAS) using standard procedures and protocols. The shoot length (SL) and root length (RL) of the selected maize seedlings from each treatment was measured using a measuring scale. The shoot fresh weight (SFW) and root fresh weight (RFW) were measured using a sensitive analytical weighing balance. Subsequently, seedlings were oven dried at 70 °C until a constant weight achieved. While, the shoot dry weight (SDW) and root dry weight (RDW) were then measured using the same sensitive analytical weighing balance.

### Observations and data collection for the field experiment

In the field experiment, plant height was measured at crop maturity using a measuring scale, from of the base of stem (ground level) to the tip of the plants. Measurements were taken from five randomly selected plants per experimental unit, and then mean value was computed. Similarly, the number of leaves per plant was recorded from these five selected plants in each experimental unit. Additionally, leaf length was measured from the base of straightened leaf (where it attaches to the stem) to the tip of leaf blade using a measuring scale by selecting three leaves from the selected five plants in each experimental unit and the mean value was computed. The chlorophyll contents of selected five plants were recorded using a chlorophyll meter (SPAD-502, Tokyo, Japan). The net leaf photosynthetic rate (A), transpiration rate (T), stomatal conductance (gs) and water use efficiency (WUE) were measured using a CIRAS-3 (80018-3 Portable Photosynthesis System). It operated at ambient 30 °C leaf temperature, photosynthetic photon-flux density at 767 µmol m^2^ s^− 1^, 97 kPa atmospheric pressure, 90 mL min^− 1^ air flow, and 325 µmol mol^− 1^ CO_2_ concentrations. These measurements were recorded from 10:30 am to 1:30 pm.

Furthermore, cob length was measured using a measuring scale on five cobs randomly selected from different plants and average was computed to determine the mean cob length. Similarly, the number of grains per cob was recorded by selecting five cobs from different plants. The grains were then separated, counted and the mean number of grains per cob was calculated. Moreover, 1000-grains weight was determined by counting thousand grains from each treatment unit and weighed them using an analytical weighing balance. Additionally, the crop was harvested from a randomly selected three-meter squares area (3 m^2^) in each experimental unit. The harvested cobs were separated and dried under sunlight. Once dried, the grains were separated and weighed using an analytical balance. The recorded grains weight was then converted into tons per hectare (t ha^− 1^) of grain yield by extrapolating the data to a 10,000 m^2^ area. Furthermore, the crop, including cobs, leaves, and stalks, was harvested from an area of 3 m^2^, and dried under sunlight. After the sun-drying, the total biomass was weighed, and the recorded weight was converted into tons per hectare (t ha⁻¹) of biological yield by scaling up to a 10,000 m² area.

### Determination of enzymatic antioxidants activity

Enzymatic antioxidant activities were measured using 0.5 g of fresh leaf samples. The leaf samples were homogenized in 5 ml of extraction buffer (50 mM Na_2_HPO_4_ pH 7.0 and 1 mM dithiothreitol) using a chilled pestle and mortar. The homogenate was then centrifuged at 9000 rpm at 4 °C for 15 min and the resulting supernatant was used for enzyme assays. The scientific protocols of H. Aebi [55], were used for the determination of enzyme activities of catalase (CAT). The reaction mixture consisted of 100 µL of enzyme extract, 100 µL of 5.9 mM H₂O₂, and 2 mL of 50 mM phosphate buffer. Moreover, superoxide dismutase (SOD) activity was estimated by measuring the enzymatic potential to obstacle photo-chemical decrease in nitro blue tetrazolium chloride (NBT). To achieve this, the reaction mixture was prepared by adding 10 µL NBT, 10 µL riboflavin, 50 µL enzyme extract, 20 µL Triton X, 80 µL distilled water, 50 µL phosphate buffer and 20 µL methionine. Furthermore, peroxidase (POD) activity was measured following the method of Kar and Mishra [56],. The reactants used were composed of 5 mL of HCL buffer (0.1 M), 5 mL pyrogallol (10 mM), 5 mM of H_2_O_2_ (5 mM) and 100 µL enzyme extract as described by Saleem et al. [57]. After preparing the reaction mixtures for CAT, SOD and POD, they were transferred into the micro-well plates and analyzed using an Enzyme-Linked Immunosorbent Assay (ELISA) instrument at wavelengths of 240 nm, 560 nm and 470 nm, respectively. The absorbance readings were then recorded.

### Statistical analysis

Statistical analysis was performed using the R statistical software v. 4.1.2 (R Core Team, 2020). Homogeneity of variance and normality were also initially checked for using Levene’s test and the Shapiro–Wilk test, respectively. To analyze the effects of heat stress and silicon seed inoculation on the growth, physiology, antioxidant activity, osmolytes production and yield traits of maize hybrids, analysis of variance (ANOVA) was performed using the ‘agricoalae’ package. All treatment means were separated through Tukey’s Honest Significant Difference (HSD) test with 1% probability level for pot experiment, and 5% probability level for field experiment [58]. For the evaluation of maize hybrids for each trait, a principal component analysis (PCA) was plotted using the package ‘ggbiplot2’ utilizing the scaling and centering feature. The statistical packages; readxl, cor and corplot were also used to assess the association between the seedling growth and physiological traits, antioxidants activity and yield components of the maize hybrids. Heatmaps was developed using the ggplot2, and heatmaps packages in R. SigmaPlot v. 15 was also used for the graphical representation of the recorded data.

## Results

### Effect of heat stress on the seedling growth and morphology of maize hybrids in glasshouse

Heat stress conditions (40 °C ± 3 and 45 °C ± 3) negatively affected the growth traits of all the studied maize hybrids as compared to the control (without heat stress) in the experiment conducted under glasshouse (Table 2). All the growth traits (SL, RL, SFW, RFW, SDW and RDW) were significantly decreased compared to the control treatment. The lower decrease in the studied growth traits was observed in the hybrids FH-1046, YH-5427 and DK-7024 under heat stress conditions. Conversely, the higher decrease was recorded in the growth traits of hybrids NK-6654, FH-949 and AA-9933 under heat stress conditions. However, the minimum reduction in the growth traits of the seedlings was recorded in maize hybrid YH-5427, while the maximum reduction was recorded in the maize hybrid AA-9633. Based on the performance of the maize hybrids, YH-5427 and AA-9633 were categorized as heat tolerant and heat sensitive, respectively, and were selected for further field study (Table 2).

**Table 2:** Effect of different heat stress levels on seedling growth traits of maize hybrids under glasshouse conditions

Treatments	SL (cm)	RL (cm)	SFW (g)	RFW (g)	SDW (g)	RDW (g)
Control						
FH-1046	76.1 b	11.9 hi	78.1 b	6.18 hi	17.5 b	1.51 hi
YH-5427	82.4 a	13.9 efg	84.6 a	7.20 efg	18.9 a	1.76 efg
NK-6654	67.3 cd	10.7 ij	69.1 cd	5.55 ij	15.5 cd	1.36 ij
AA-9933	63.1 de	9.3 j	64.8 de	4.83 j	14.5 de	1.18 j
FH-949	69.4 c	10.9 i	71.3 c	5.67 i	15.9 c	1.39 i
DK-7024	67.7 cd	12.5 gh	69.5 cd	6.49 gh	15.5 cd	1.59 gh
**40 °C ± 3**						
FH-1046	62.4 de	15.0 de	64.1 de	7.80 de	14.3 de	1.91 de
YH-5427	69.5 c	16.7 c	71.3 c	8.67 c	15.9 c	2.12 c
NK-6654	53.6 fgh	12.9 fgh	55.0 fgh	6.69 fgh	12.3 fgh	1.63 fgh
AA-9933	49.6 hi	12.0 hi	51.0 hi	6.20 hi	11.4 hi	1.51 hi
FH-949	58.2 ef	14.0 ef	59.8 ef	7.27 ef	13.4 ef	1.78 ef
DK-7024	59.4 ef	14.3 ef	61.0 ef	7.42 ef	13.6 ef	1.81ef
**45 °C ± 3**						
FH-1046	49.5 hi	18.3 b	50.9 hi	9.50 b	11.4 hi	2.32 b
YH-5427	57.6 efg	19.9 a	59.2 efg	10.29 a	13.2 efg	2.51 a
NK-6654	44.5 ij	16.2 cd	45.7 ij	8.41 cd	10.2 ij	2.05 cd
AA-9933	38.7 j	15.2 de	39.7 j	7.88 de	8.9 j	1.93 de
FH-949	45.4 i	16.7 c	46.6 i	8.67 c	10.4 i	2.12 c
DK-7024	52.0 gh	16.3 cd	53.4 gh	8.46 cd	11.9 gh	2.07 cd
HSD (*p* ≤ 0.01)						
H	**	**	**	**	**	**
HS	**	*	*	**	**	*
H × HS	*	*	*	*	*	*

* = significant at *p* ≤ 0.05 and ** is significant at *p* > 0.01, H = Maize hybrids; HS = Heat Stress levels

### Effect of heat stress and Si inoculation on physiological traits of maize hybrids in field conditions

**Fig. 4:**
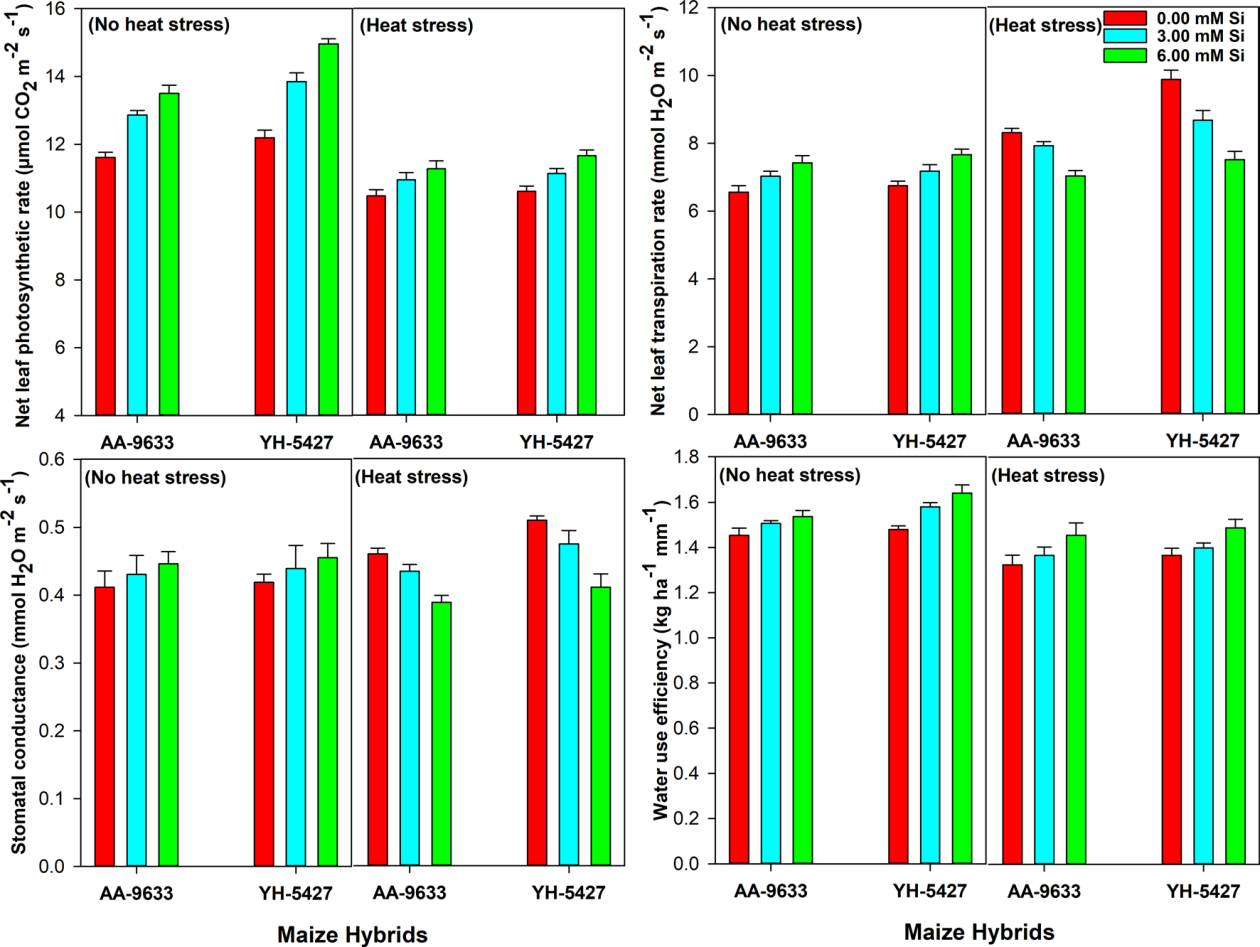
Effect of silicon seed inoculation on physiological traits (photosynthetic rate, stomatal conductance, transpiration rate and water use efficiency) of maize hybrids under heat stress conditions in field study

Heat stress significantly affected the physiological traits, including net leaf photosynthetic rate (A), stomatal conductance (gs), net leaf transpiration rate (T), and water use efficiency (WUE) in maize crop during the field study. However, silicon seed inoculation improved these physiological traits under heat stress conditions (Fig. 4). Moreover, the heat stress significantly enhanced gs and T in maize crop; however, silicon inoculation mitigated these effects by reducing gs and T in maize crop under heat stress conditions. Additionally, maize hybrids showed varying responses to Si seed inoculation under heat stress. The maize hybrid YH-5427 demonstrated superior physiological traits with 6 mM Si seed inoculation under heat stress compared to the control (without Si inoculation). Similarly, silicon seed inoculation also improved the physiological traits of maize hybrid (AA-9633) under heat stress conditions (Fig. 4).

### Effect of heat stress and Si inoculation on antioxidants activity and protein contents of maize hybrids in field conditions

**Fig. 5:**
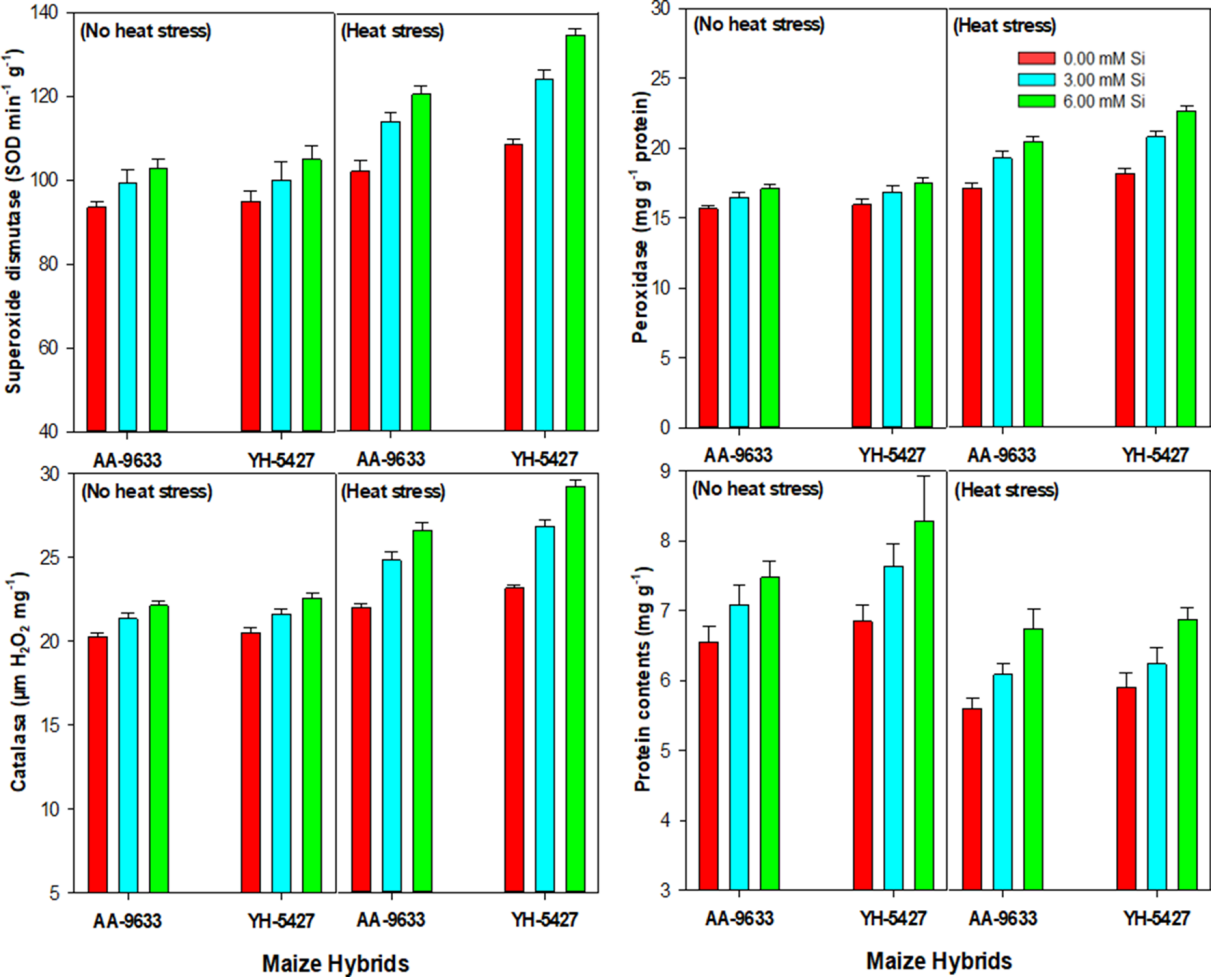
Effect of silicon seed inoculation on the antioxidant’s activity of maize hybrids under heat stress conditions in field study

The activity of antioxidant enzymes, including superoxide dismutase (SOD), peroxidase (POD), and catalase (CAT), increased significantly under heat stress conditions. Whereas, the protein contents decreased markedly under heat stress conditions as compared with control (no heat stress). However, silicon (Si) seed inoculation mitigated these effects by enhancing both antioxidants activity and protein contents under heat stress conditions (Fig. 5). The response to Si seed inoculation varied among maize hybrids. The maximum increase in antioxidant’s activity was recorded in maize hybrid YH-5427 with the seed inoculation of Si at 6 mM under heat stress, compared to the control (without Si inoculation). Similarly, the silicon seed inoculation also enhanced antioxidants activity in maize hybrid AA-9633 under heat stress conditions (Fig. 5).

### Effect of heat stress and Si inoculation on growth traits of maize hybrids in field conditions

**Fig. 6:**
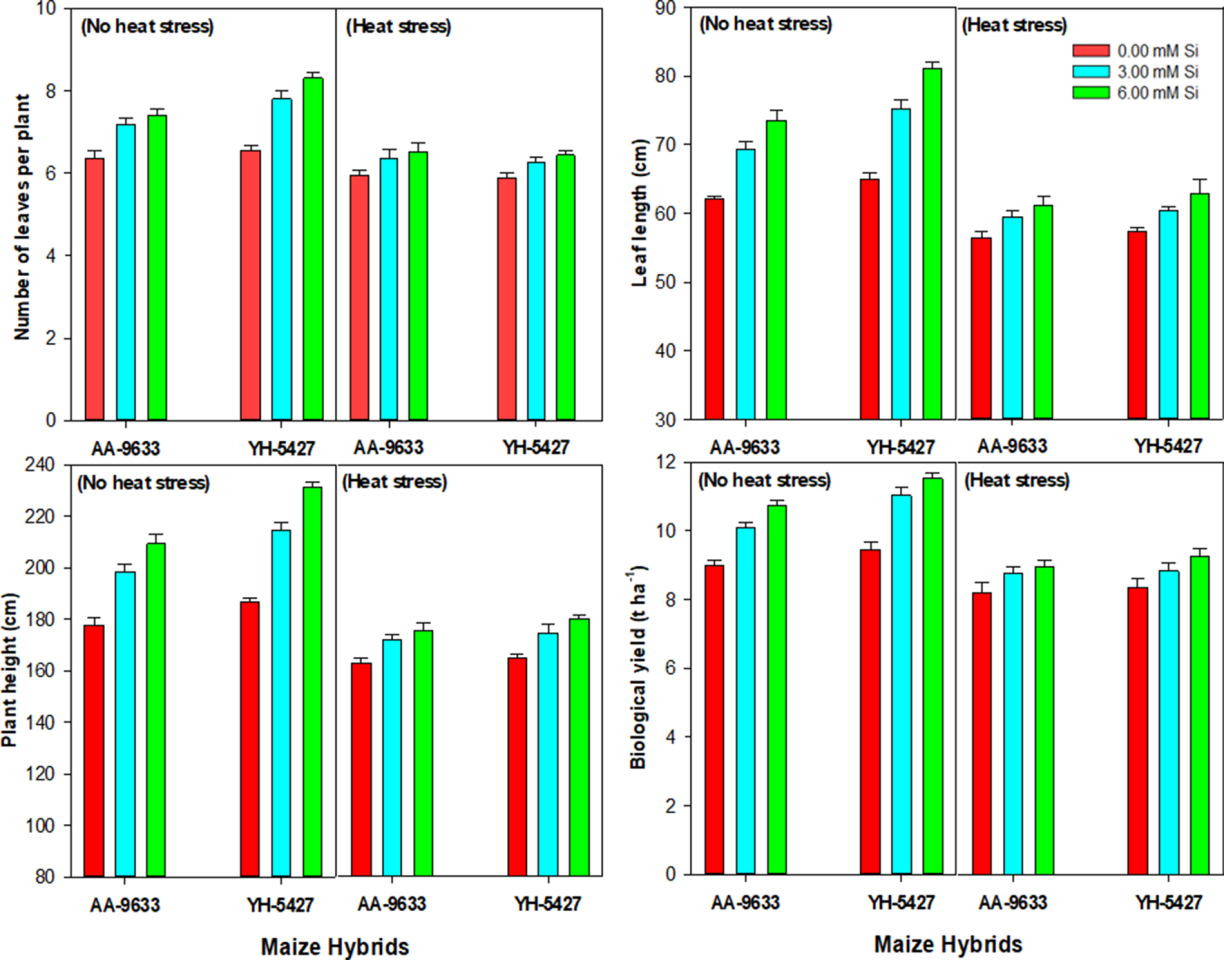
Effect of silicon seed inoculation on the growth traits and biological yield of maize hybrids under heat stress in field conditions

Heat stress also significantly reduced plant growth parameters including number of leaves per plant (NLPP), leaf length (LL), plant height (PH) and biological yield in maize crop. In contrast, silicon (Si) seed inoculation markedly improved these growth traits and biological yield under heat stress conditions compared with no heat stress (Fig. 6). The superior growth traits and higher biological yield were observed in maize hybrid YH-5427 with the seed inoculation of Si at 6 mM under heat stress as compared to control (without Si inoculation). Similarly, silicon seed inoculation also enhanced growth traits and biological yield in maize hybrid AA-9633 under heat stress conditions (Fig. 6).

### Effect of heat stress and Si inoculation on yield traits of maize hybrids under field conditions

**Fig. 7:**
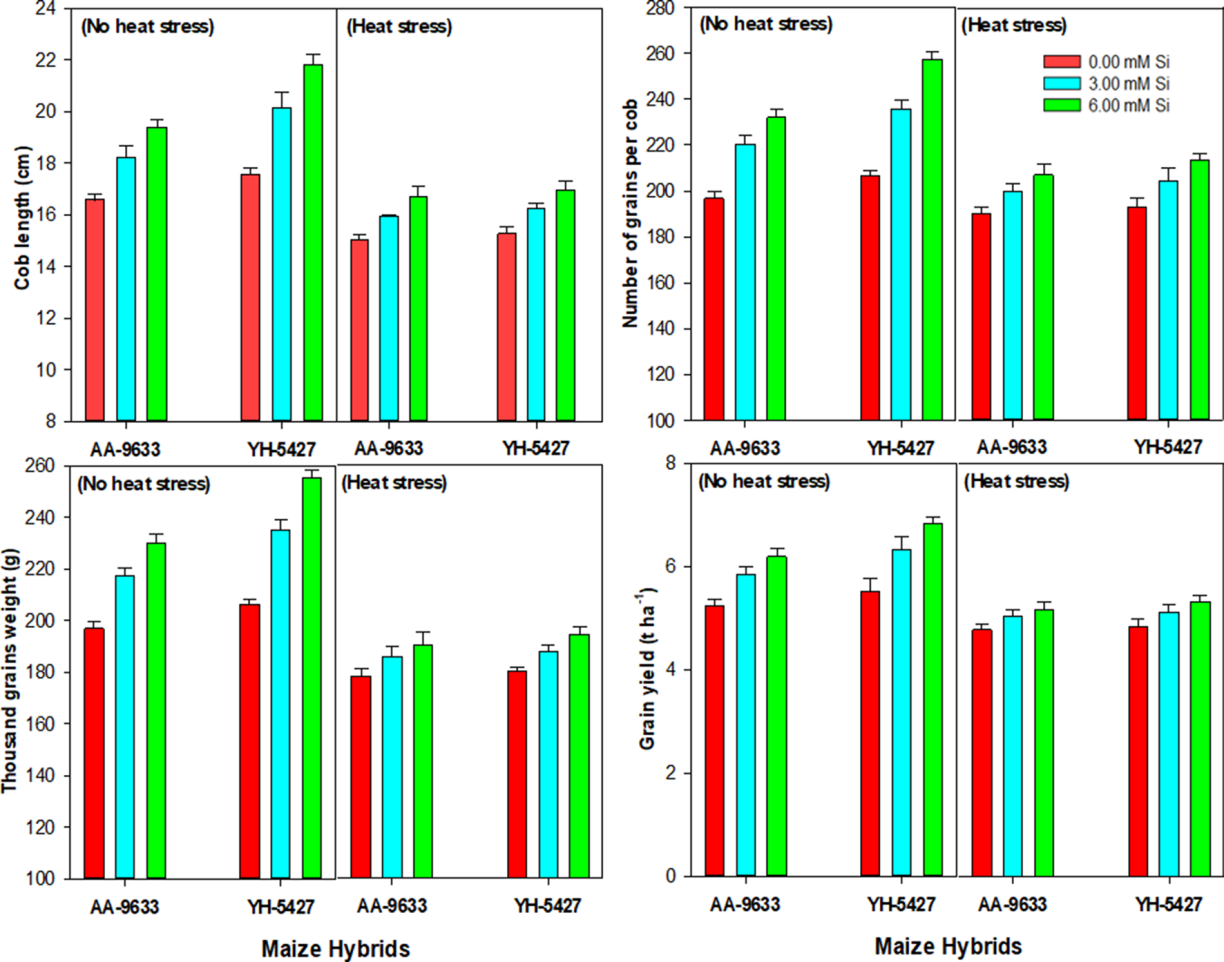
Effect of silicon seed inoculation on the yield traits and grain yield of maize hybrids under heat stress in field conditions

Yield traits including cob length (CL), number of grains per cob (NGPC), thousand grain weight (TGW) and overall grain yield of the maize crop were also significantly reduced under heat stress conditions, showed the negative impacts of elevated temperature (heat stress) on maize productivity. Heat stress affected key physiological processes, grain formation, seed filling and overall maize crop yield. However, silicon seed inoculation effectively mitigated these negative effects, leading to significantly improvements in yield traits and grain yield in maize under heat stress conditions (Fig. 7). Among the studied maize hybrids, YH-5427 showed better resilience to heat stress when inoculated with 6 mM-Si, by revealing more cob length, enhanced number of grains per cob, better thousand grain weight and finally improved the grain yield compared to the control treatment (without Si inoculation). It indicates that silicon plays a crucial role in enhancing stress tolerance mechanisms, possibly by improving antioxidant defense, and photosynthetic efficiency under heat stress conditions. Similarly, maize hybrid AA-9633 also demonstrated improvements in yield traits and grain yield with silicon seed inoculation under heat stress conditions but lower compared with the YH-5427 (Fig. 7). These findings revealed the potential of silicon seed inoculation and also prove it as an effective agronomic mitigation strategy to minimize the negative effects of elevated temperature and further improve maize productivity in heat stress environments.

### Correlation analysis

**Fig. 8:**
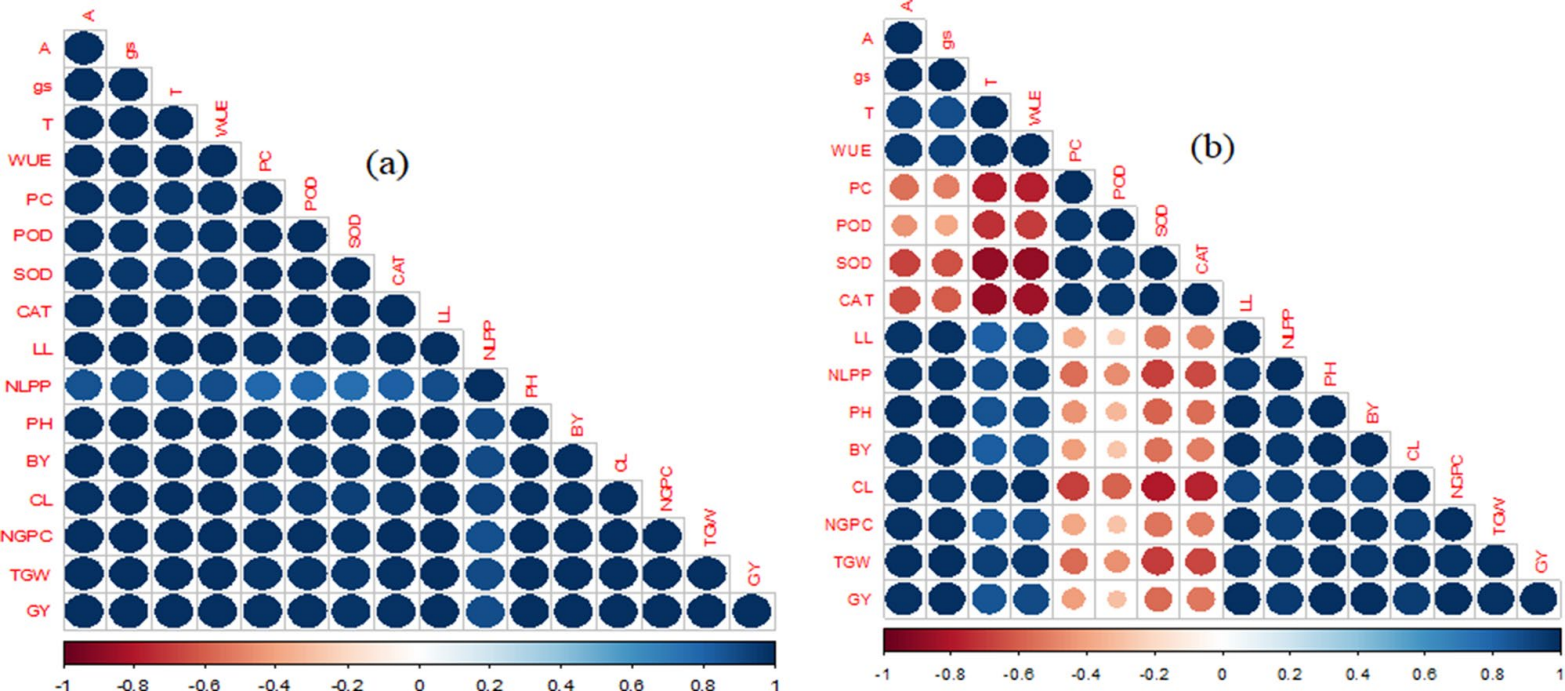
The correlation map indicates the effect of silicon seed inoculation on the growth traits and biological yield of maize hybrids under control (no heat stress) (**a**) and heat stress (**b**) conditions

The physiological traits of maize hybrids showed a positive correlation with each other and with growth and yield traits under both no heat stress and heat stress conditions (Fig. 8a, b). Similarly, the physiological traits also showed a positive correlation with antioxidants activity under no heat stress conditions. However, there was a weak and negative correlation observed between photosynthetic rate and stomatal conductance and antioxidants activity under heat stress conditions. Furthermore, a highly negative correlation found between water use efficiency and transpiration rate and antioxidants activity under heat stress conditions. The growth traits of maize hybrids indicated a positive correlation with each other and with physiological and yield traits under both no heat stress and heat stress conditions (Fig. 8a, b).

However, in heat stress conditions, there was a weak and negative correlation between leaf length, plant height and biological yield of maize hybrids with peroxidase activity Additionally, there was a highly negative correlation between the number of leaves per plant, protein contents, superoxide dismutase and catalyse under heat stress conditions. All the yield traits also showed a positive correlation with eachother, and also with growth, physiological and yield traits of maize hybrids under both no heat stress and heat stress conditions. However, there was a weak and negative correlation between yield traits and antioxidants activity, except for cob length which showed a highly negative correlation with protein contents, superoxide dismutase and catalyse activity in the maize hybrids (Fig. 8a, b).

The traits are defined as follows: A = Photosynthesis rate (µmol CO_2_ m^− 2^ s^− 1^); gs = Stomatal conductance (mmol H_2_O m^− 2^ s^− 1^); T = Transpiration rate (mmol H_2_O m^− 2^ s^− 1^); WUE = Water use efficiency (kg ha^− 1^ mm^− 1^); PC = Protein contents (mg g^− 1^); CAT = Catalase (µmol min^− 1^ mg^− 1^ protein); SOD = Superoxide dismutase (µmol min^− 1^ mg^− 1^ protein); POD = Peroxidase (µmol min^− 1^ mg^− 1^ protein); LL = leaf length (cm); NLPP = Number of leaves per plant; PH = Plant height (cm); BY = Biological yield (kg ha^− 1^); CL = Cob length (cm); NGPC = Number of grains per cob; TGW = Thousand grains weight (g) and GY = Grain yield (kg ha^− 1^). The blue color indicates a positive correlation, while the brown and dark brown color indicate a negative correlation. The size of the circle represents the strength of the association among traits.

### Grouped heat map visualization for the effect of silicon seed inoculation on maize hybrid under heat stress conditions

**Fig. 9:**
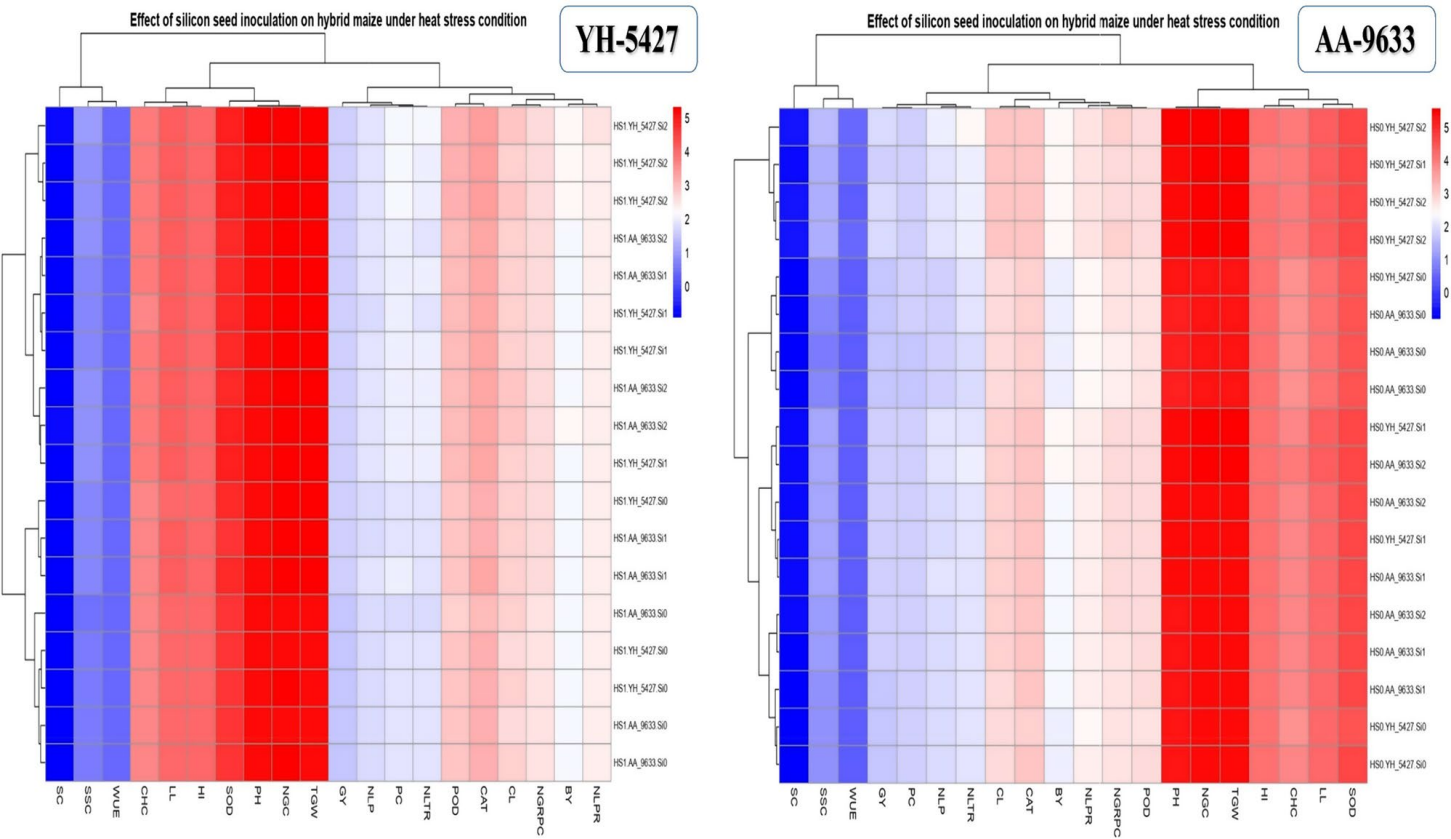
Heat map analysis indicating the role of silicon levels on the morpho-physiological traits, antioxidants activity and yield components of maize hybrids under heat stress conditions

These heat maps indicate the effect of different levels of silicon on the morpho-physiological traits, antioxidants activity and yield components of maize hybrids under heat stress. In these maps, the rows represent maize hybrids and silicon levels, whereas the columns are indicating the measured morpho-physiological traits, antioxidants activity and yield components of maize hybrids under heat stress conditions. The color gradients, transitioning from red (indicating higher values) to blue (indicating lower values), represents the long-transformed values of the measured traits in maize hybrids (Fig. 9). Additionally, the hierarchical clustering of both rows and columns illustrates patterns of similarity, grouping maize hybrids and silicon levels based on their performance profiles, as well as traits with related functions (Fig. 9). The heat map analysis uncovers potential correlations among various crop attributes, helping to identify maize hybrids with enhanced stress tolerance and offering valuable insights into silicon’s critical role in alleviating heat stress effects. The red color gradient represents the highest increase in measured traits and improved stress tolerance with different silicon levels, while the blue gradient signifies the least improvements in maize hybrid traits under heat stress (Fig. 9).

## Discussion

Heat stress is one of the most disturbing threats to maize productivity in arid and semi-arid regions [59]. When temperature rises beyond an optimum level for a specific period of time, it leads to heat stress in the plants and adversely affects the growth and development [7, 60]. Heat stress inhibits the growth and yield traits of maize crop by disturbing the physiological processes, enzyme inactivation, pollen abortion and silks desiccation [61, 62]. In severe heat stress conditions, production of reactive oxygen species (ROS), electrolyte leakage and enhanced malondialdehyde contents leads to oxidative stress, and cell membrane injury [18, 61]. Moreover, the heat stress shortens the phenological stages of maize crop by early accumulation of heat units (growing degree days), resulting in stunted growth and impaired metabolic processes. However, heat tolerant germplasm has greater ability to minimize the adverse effects of heat stress [63]. These tolerant plants have evolved different avoidance and heat tolerance mechanisms by regulating the physiological processes, protein synthesis, and antioxidants activities [64].

In the glass house study, heat stress significantly decreased the morpho-physiological and growth traits of all the studied maize hybrids. It is reported that temperature above the normal range causes osmotic and oxidative stress, which leads to decreased physiological traits in maize hybrids. According to Ayub et al. [65], temperature above 35 °C adversely affect the growth and physiological traits in maize. The current study also demonstrated a decreasing trend in the morpho-physiological traits of the maize hybrids, with these traits showing significant differences under heat stress conditions. Moreover, it has been reported that heat stress leads to chlorophyll contents degradation, which limits photosynthetic performance in the heat stressed plants [66], a similar observation found in the current study. Furthermore, it is reported that heat tolerant maize hybrids have greater chlorophyll contents and ability to maintain photosynthetic activity and other physiological traits than heat sensitive [67]. Consequently, maize hybrid “YH-5427” showed comparatively higher values of morpho-physiological traits in comparison with other studied maize hybrids. In contrast, maize hybrid AA-9633 showed the lowest values of morpho-physiological traits under heat stress conditions and was categorized as drought sensitive. Thus, YH-5427 has a greater potential to alleviate the adverse effects of high temperature by maintaining physiological activity under heat stress conditions, thereby being marked as a heat tolerant maize hybrid.

Similarly, in field conditions, heat stress significantly decreased the physiological, antioxidants activity, growth and yield traits in maize hybrids. It has been demonstrated by Meseka et al. [68] that heat stress causes chlorophyll contents degradation, and ultimately lowers the photosynthetic activity. Moreover, an optimum temperature (28–31 °C) is required to the maize plants for proper functioning of the photosynthetic process. Therefore, when temperature rises above the normal range leads to osmotic and oxidative stresses, decreasing the physiological processes in maize. Similar results were also observed in the present study that corresponds with previous studies of Hussain et al. [66] and Sabagh et al. [67]. However, the physiological traits were improved with silicon seed inoculation of 6 mM under heat stress conditions. These improvements in the physiological traits were due to the imperative role of Si in improving the photosynthetic activity in the crop plants under heat stress [64, 69]. It is reported that silicon also plays a vital role in scavenging ROS production by improving the enzymatic antioxidant activities. These antioxidants protect the cell from thermal injury, which in turn maintains proper functioning of the cells and resulting in higher physiological traits in the plants [70, 71]. The Si seed inoculation support plants to absorb more amount of water and maintains relative water contents in the leaves to promote leaves turgidity [18, 72].

Moreover, the antioxidant activities are increased in plants under heat stressed conditions when compared to normal sown maize. While, the protein contents are decreased when the crop is exposed to heat stress conditions. Whereas, the results showed that heat tolerant maize hybrid maintained the highest protein contents under heat stress conditions. It might be correlated with the inherent ability of plants to maintain protein contents, and enhance peroxidase, superoxide dismutase and catalase activities under stress conditions [61]. Moreover, the Si seed inoculation further improved the activities of enzymatic antioxidants under heat stress conditions. Though, the maximum increase in the activities of SOD, POD, CAT and protein contents was recorded with the seed inoculation of 6 mM Si. It might be due to vital role of Si in the regulation of metabolic processes and lowering the ROS production under heat stress conditions. The current research findings are in an agreement with previous recent studies of Meseka et al. [69], who reported that Si application improves the enzyme activities and non-enzymatic antioxidants under heat stress conditions. It has also been reported by Kumar et al. [15] where Si application improved peroxidase activities, superoxide dismutase and catalase under heat stress, which also supports the current study findings. Thus, improved antioxidants activity helps the plants to reduce the cellular damage and ultimately improves the growth and yield traits in maize [71, 73].

The current study revealed that heat stress significantly reduced the number of leaves per plant, leaf length, plant height and biological yield in maize hybrids. This decrease in the growth traits and biological yield might be due to poor physiological and antioxidant activities under heat stress conditions, as were recorded in the present study. Additionally, the reduction in these growth traits might be associated with impaired cell division and cell elongation due to the crop exposure in heat stress [74]. However, silicon seed inoculation (6 mM) improved the number of leaves per plant, leaf length, plant height and biological yield under heat stress as compared control treatment. This improvement may be correlated with the improved physiological traits due to Si seed inoculation under heat stress [18]. Furthermore, higher crop growth and yield traits might be associated with the fact that Si application improves ROS detoxification by increasing enzymatic activities and antioxidants under heat stress [64]. Moreover, the maximum cob length, number of grains per cob, thousand grain weight and grain yield were recorded with the seed inoculation (Si at 6 mM). It might be because of improved physiological traits under heat stress as observed in the present study. Several previous studies have also reported that Si application improves the grain yield and yield components of the plants under stress conditions [75, 76].

Additionally, there is a genetic variation also exits among maize hybrids to maintain the physiological processes and antioxidants activity to efficiently utilize higher temperatures for increasing their growth and development [77, 78]. Therefore, heat tolerant maize hybrid (YH-5427) showed good physiological traits and antioxidants activities in comparison to heat sensitive (AA-9633) maize hybrid. Thus, higher expression of antioxidants in heat tolerant maize hybrids (YH-5427) resulted in higher physiological, growth and yield traits under heat stress conditions [32]. Hence, the analysis of expression of antioxidants in the heat tolerant maize hybrids could be used to identify the heat tolerant maize germplasm. Based on correlation results, all the physiological, antioxidants activity, growth and yield traits were also positively correlated with each other under no heat stress conditions. However, antioxidants activity was negatively correlated with growth, physiological and yield traits under heat stress conditions. This negative correlation might be due to increase in antioxidants activity and decrease in the growth, physiological and yield traits in maize hybrids under heat stress.

Overall, the findings showed that high grain yield in maize hybrids was recorded with the seed inoculation (Si at 6 mM) due to better growth, physiological and antioxidants activity. These findings highlight the potential of silicon (Si) seed inoculation (6 mM) in maize hybrid (YH-5427) as a practical and cost-effective strategy for mitigating heat stress, thereby improving crop resilience and yield stability under high-temperature conditions. Given its ease of application, low cost, and compatibility with existing agronomic practices, Si seed treatment is highly suggested for farmers in heat-stressed regions as a sustainable approach to enhance maize production and ensure food security amidst climate change challenges.

## Conclusion

The study explored the heat stress mitigation strategies in maize crop at reproductive phases via using Si seed inoculation and maize hybrids under glass house and field conditions. The effect of different Si seed inoculations was demonstrated on the physiology, growth, antioxidants activity and yield traits of the selected maize hybrids (sensitive and tolerant) under heat stress conditions in the field. The maize hybrids varied in their response to heat stress, and the heat tolerant maize hybrid “YH-5427” showed the maximum physiology, growth, antioxidants activity and yield traits and consequently the highest grain yield with the seed inoculation of Si @ 6 mM under heat stress. Therefore, based on the findings, the sowing of maize hybrid (YH-5427) with Si seed inoculation at 6 mM emerged as a sustainable strategy for mitigating the adverse impacts of heat stress, enhancing crop resilience and overall productivity. This approach can be seamlessly integrated into existing agricultural practices without requiring significant modifications to conventional seed treatment protocols. It would be recommended to the farmers as Si seed inoculation not only improves heat tolerance but also complements traditional fertilization strategies, particularly in Si-deficient soils, where its application can further enhance plant health. Moreover, future studies are needed to elaborate the effects of Si seed inoculation on the physiology, growth and yield traits subjected to combined heat and drought stress conditions under multi-environmental conditions and multi-year field experimentation.

## Data Availability

All the data related to this work can be sourced from the corresponding authors.
